# Mass Spectrometry-Based Identification of Bioactive Bee Pollen Proteins: Evaluation of Allergy Risk after Bee Pollen Supplementation

**DOI:** 10.3390/molecules27227733

**Published:** 2022-11-10

**Authors:** Eliza Matuszewska, Szymon Plewa, Dagmara Pietkiewicz, Kacper Kossakowski, Joanna Matysiak, Grzegorz Rosiński, Jan Matysiak

**Affiliations:** 1 Department of Inorganic and Analytical Chemistry, Poznan University of Medical Sciences, 3 Rokietnicka Street, 60-806 Poznań, Poland; 2 Faculty of Health Sciences, Calisia University, 13 Kaszubska Street, 62-800 Kalisz, Poland; 3 Department of Animal Physiology and Development, Faculty of Biology, Adam Mickiewicz University in Poznan, 6 Uniwersytetu Poznańskiego Street, 61-614 Poznań, Poland

**Keywords:** allergens, bee pollen, MALDI-TOF, mass spectrometry, proteomics

## Abstract

Bee pollen, because of its high content of nutrients, is a very valuable medicinal and nutritional product. However, since its composition is not completely studied, the consumption of this product may cause adverse effects, including allergic reactions. Therefore, this study aimed to discover and characterize the bioactive proteins of bee pollen collected in Poland, focusing mainly on the allergens. For this purpose, the purified and concentrated pollen aqueous solutions were analyzed using the nanoLC-MALDI-TOF/TOF MS analytical platform. As a result of the experiments, 197 unique proteins derived from green plants (*Viridiplantae*) and 10 unique proteins derived from bees (*Apis* spp.) were identified. Among them, potential plant allergens were discovered. Moreover, proteins belonging to the group of hypothetical proteins, whose expression had not been confirmed experimentally before, were detected. Because of the content of bioactive compounds—both beneficial and harmful—there is a critical need to develop guidelines for standardizing bee pollen, especially intended for consumption or therapeutic purposes. This is of particular importance because awareness of the allergen content of bee pollen and other bee products can prevent health- or life-threatening incidents following the ingestion of these increasingly popular “superfoods”.

## 1. Introduction

The demand for natural products is growing due to their nutritional and potentially therapeutic properties [1]. Compounds from biological sources are used in phytotherapy, diet, and cosmetology. The natural products include bee products such as honey, propolis, beeswax, royal jelly, and bee pollen. The use of bee honey for the prevention and treatment of infectious diseases, or propolis as an antibacterial, antifungal, and antiviral agent, is well known [2,3,4,5]. Moreover, bee pollen—a subject of this study—is a valuable source of proteins, essential amino acids, carbohydrates, lipids, bio-elements, and vitamins [6,7]. Thanks to its extraordinarily rich chemical composition, bee pollen can be used as a dietary supplement [8]. This product can enhance the defense system [9]. That is why it is recommended as a regenerative nutrient for the elderly, children, and for people whose lifestyle is active. It is also willingly used as a supportive food for recovery. Bee pollen may be eaten directly as a raw material or packed in capsules or other drug forms.

Bee pollen is a complex product classified into the superfood category and used in pharmaceutical industries [10]. However, its composition is still not fully investigated. Therefore, this paper focuses on the proteomic composition of bee pollen. In botanical terms, it is a mixture of flower pollen collected by bees from different plant species and to which bees add enzymes (i.e., amylase) secreted by salivary glands, as well as nectar, honey, or wax. Small pellets of bee pollen are transported in the pollen basket of bees’ legs to the beehive, where they are stored and used as a primary protein source for the bee colony [11]. The plant source, geographical origin, soil type, climatic conditions, and bee breed determine bee pollen composition [12]. Moreover, the pollen’s color ranges from bright yellow to dark brown or black. It depends on the composition of lipidic dyes or pigments from flower anthers and the botanical taxonomy of the plant source [6]. The interspecific difference affects the qualitative and quantitative composition of pollen bioactive compounds, nutrients, and minerals. Therefore, the composition of bee pollen from different botanical sites should be thoroughly investigated. In addition, guidelines for standardizing bee pollen for use as a food supplement should be developed. Moreover, since bee pollen is a multi-active product of natural origin, studies on its safety are strongly needed [13].

Bee pollen consists of sugars, polysaccharides, starch, and fibers. In addition, it contains lipids, minerals, and a wide range of secondary plant metabolites (i.e., phytosterols, anthocyanins, vitamins, polyphenols, etc.) [14]. The primary macronutrients of bee pollen are proteins. Bee pollen contains all functional proteins composed of essential and nonessential amino acids [6]. However, the available literature reports do not clearly describe the exact types of proteins. Therefore, we attempted to identify new proteomic compounds present in bee pollen harvested in west-central Poland. This study aimed to perform an in-depth proteomic analysis of bee pollen with particular emphasis on identifying allergens and evaluating the risk of allergic reactions after consumption of this product.

Bee pollen can ensure a good food substitute and has potential therapeutic usage. However, its consumption also may be associated with the risk of side effects, such as allergic reactions and molds, mycotoxins, pesticides, and toxic metals poisoning [15,16]. In order to prove the safety of its consumption, it is necessary to examine the composition of bee pollen in terms of potential allergens, which are generally proteins. Therefore, in the presented study, we performed a proteomic analysis of four different color bee pollens from *Apis mellifera* using a MALDI-TOF (matrix-assisted laser desorption/ionization-time of flight) mass spectrometer. We discovered potential plant allergens and showed differences between the individual colors of the bee pollen.

## 2. Results

A total number of 207 unique proteins was identified in all bee pollen samples. The proteins were taxonomically classified into *Viridiplantae* (green plants) clade (n = 197) and *Apis* spp. bees (n = 10). Only the identification of proteins based on at least three peptides was considered reliable (Supplementary Materials, Table S1).

### 2.1. Proteins Taxonomically Classified to Viridiplantae Clade

**Figure 1:**
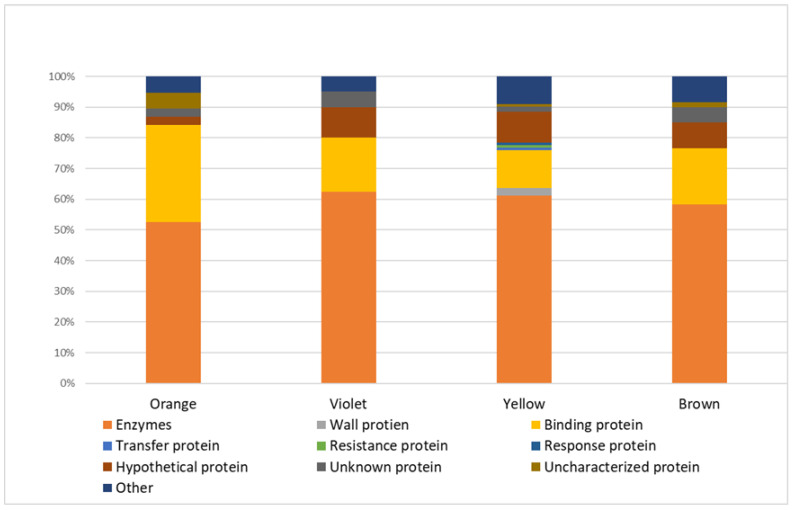
The percentage of identified proteins with a specific function in bee pollen of a given color.

In a group of *Viridiplantae* proteins, enzymes were the most numerous functional class (Figure 1). In all samples, 129 plant enzymes (≈65% of all proteins) were identified, including:

- n = 74 in yellow bee pollen (≈61% of all proteins identified in yellow pollen);
- n = 35 in brown bee pollen (≈58% of proteins identified in brown samples);
- n = 25 in violet bee pollen (≈63% of proteins identified in violet pollen);
- n = 20 in orange bee pollen (≈53% of proteins identified in orange samples).

The next abundant functional group was binding proteins (n = 22, standing for ≈11% of proteins identified in all samples):

- n = 15 in yellow bee pollen (≈12% of all proteins identified in yellow pollen);
- n = 12 in orange bee pollen (≈32% of proteins identified in orange pollen);
- n = 11 in brown bee pollen (≈18% of proteins identified in brown pollen);
- n = 7 in violet bee pollen (≈18% of proteins identified in violet pollen).

Another functional class of green plant proteins identified in bee pollen was wall (n = 3), transfer (n = 1), response (n = 1), and resistance (n = 1) proteins.

Moreover, 17 proteins (≈9% of all proteins) were identified as hypothetical proteins. The amino acid sequence of hypothetical proteins corresponds to genetic information, but no experimental data proves their existence. Additionally, nine uncharacterized or unknown proteins (≈5% of all identified proteins) were detected. These proteins require further research to elucidate their functions and bioactivities.

**Figure 2:**
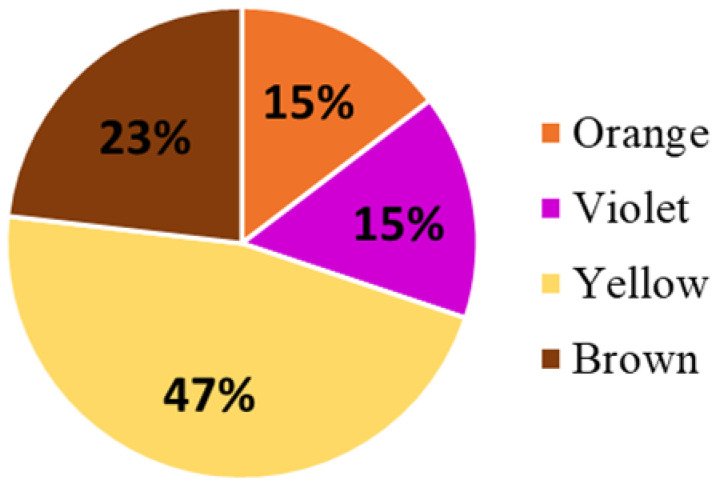
Numbers of proteins identified based on three or more peptides in bee pollen according to their color.

The number of proteins identified depended on the color of the bee pollen samples. The highest number of proteins (n = 121; 47% of all *Viridiplantae* proteins) was identified in samples of yellow pollen. In samples of brown bee pollen, 60 proteins were identified (standing for 23% of identified *Viridiplantae* proteins). The number of proteins reliably identified in violet and orange bee pollen samples was n = 40 and n = 38 (≈15% of *Viridiplante* proteins), respectively (Figure 2).

### 2.2. Proteins Taxonomically Classified to Apis Spp.

In addition to the proteins taxonomically classified as *Viridiplantae*, 10 proteins from bees were also identified (Table 1). The identified proteins are involved in carbohydrate metabolism, as well as in nutrition and development [17,18,19,20].

Based on the number of unique peptides for protein identification, the most abundant bee proteins in bee pollen are major royal jelly proteins (MRJPs), especially major royal jelly protein 1 precursor. MRJP1 was identified in all analyzed samples based on 82 peptides in orange bee pollen, 57 peptides in violet samples, 40 peptides in yellow pollen, and 39 peptides in brown samples. In addition to MRJPs, the main protein identified in the samples is glucose oxidase, identified based on 15 different peptides in violet samples, 6 peptides in yellow and brown samples, and 5 peptides in orange samples.

**Figure 3:**
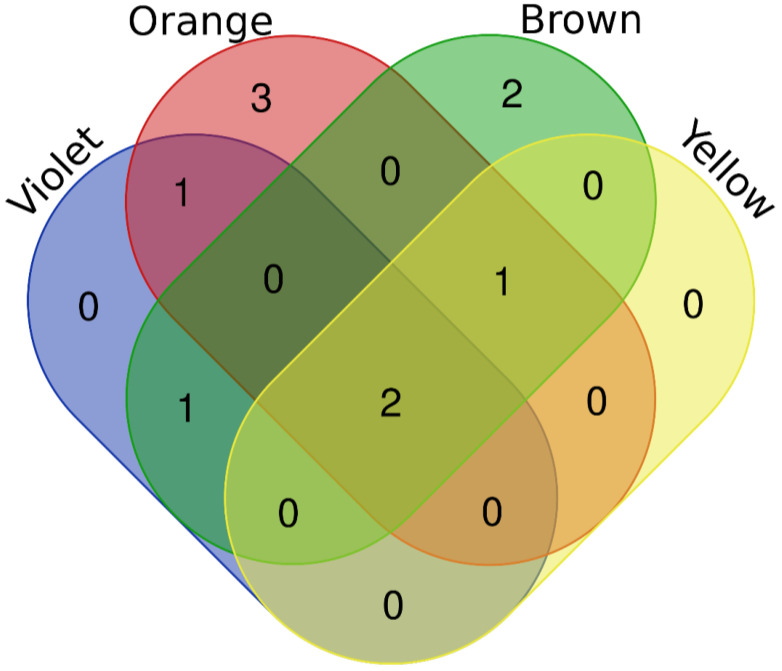
The number of *Apis* proteins identified in bee pollen samples of a given color.

Five of the *Apis* proteins were identified only in one color sample. Two proteins were identified in samples of two colors, one protein in samples of three colors, and two proteins in all bee pollen samples (Figure 3).

### 2.3. The Total Quantitative Protein Content of Bee Pollen, Based on Bradford’s Analysis

Four bee pollen aqueous solutions samples were analyzed using the Bradford method for total protein. The samples were collected in June and July 2018. The results show that the average protein concentration in the bee pollen solutions is 0.82685 mg/mL (Table 2.)

## 3. Discussion

The composition of bee pollen still needs to be studied to discover the compounds responsible for its beneficial impact on human health. Although the product has been widely used as food beneficial to the human body for years, the complexity of this bee product is still not thoroughly investigated. However, despite knowing its potential actions, the American Food and Drug Administration (FDA) has not yet approved bee pollen as an effective medication for treating any medical condition. Thus far, it is only marketed as a dietary supplement or food ingredient. This is likely due to the lack of precise compositional standardization and the uncertain quality, affecting its safe use [15]. Bee pollen may contain various harmful substances, such as heavy metals and pesticides [21,22,23]. This product is also vulnerable to microbiological contamination [24,25].

Additionally, bee pollen is poorly tested for allergens content. It contains pollen or fungal cross-reactive allergens that, in the worst case, can even lead to anaphylaxis [26,27]. It is estimated that flower pollen, which is the main component of bee pollen, can cause allergic reactions in 30% of the industrialized countries’ population [28]. That is why it is essential to develop indications for the standardization of bee pollen composition and apply them in manufacturing dietary supplements and other products based on this complex product. In order to meet these objectives, it is necessary to better understand the chemical composition of bee pollen. Therefore, this study aimed to investigate the protein composition of its aqueous solutions. The results may improve the safety of bee pollen usage, especially regarding allergen content.

As a result of our analyses, above 200 proteins were identified in bee pollen samples based on at least three unique peptides. Most proteins (n = 121) were identified in samples of yellow color. Sixty proteins were reliably identified in samples of brown pollen, and n = 40 and n = 38 proteins were identified in violet and orange samples, respectively. Such a significant number of proteins suggests the high protein content of bee pollen. To confirm this, we performed a quantitative proteomic analysis of bee pollen aqueous solutions using the Bradford method. The total protein concentration averaged 0.82685 mg/mL in the analyzed samples. However, the extraction efficiency was not tested in this study, but the total protein content of the raw material should be expected to be higher. This issue requires further analysis. According to the available literature, the mean percent protein content in bee pollen is about 17.6–22.7% [8,29]. Nevertheless, it should be underlined that analytical studies of bee pollen samples from different countries indicate variability in its composition, determined, among other things, by botanical origin or climatic conditions [6].

The most abundant group, standing for about 65% of the proteins identified in this study, were enzymes. Pollen enzymes are essential for the rendering of stored food when pollen germinates. They are crucial in facilitating the pollen tube passage through the pistil and stimulating embryo development and ovary maturation [30]. Although necessary for plants, some enzymes can harm humans when ingested. Enzymes are sensitizers that may cause respiratory allergic symptoms, including asthma and rhinitis [31]. Some allergens belong to proteolytic enzymes. The reactions they drive may be specifically dangerous, as they are released quickly, in a few minutes, from the pollen grain after moisturizing [32]. Although our analyses did not identify enzymes with known allergenic properties, it may be assumed that such particles are present in bee pollen. The functions and bioactivities of many proteins, not just enzymes, are not yet known. The goal of modern proteomics and future research will be to elucidate the role of these proteins in the human body.

Another abundant functional class of the identified proteins was binding proteins. Within this group, the most identified proteins were members of the calmodulin family. In plants, calmodulins participate in calcium signaling; thus, these proteins are crucial for development and environmental adaptation. Proper regulation of calcium metabolism also helps the plant defense microorganisms [33,34]. Although essential for plants, calmodulins may trigger allergic sensitization in humans, i.e., asthma and allergic rhinitis [35,36]. The pollen of the vast majority of plants has not yet been tested. However, it can be assumed that calmodulin, found in many species, acts analogously as an allergen. Our study identified calmodulin in all four analyzed colors of bee pollen. Therefore, allergy sufferers should be cautious when consuming this bee product, regardless of its source.

Within the group of binding proteins, we also identified an allergen protein called profilin. This actin-binding protein inhibits the polymerization of actin and promotes F-actin elongation. Profilin also participates in the membrane-cytoskeleton interaction [37]. The allergenic properties of profilin were previously confirmed [38,39]. According to the WHO/IUIS Allergen Nomenclature Home Page (https://www.allergen.org/, accessed on 2 September 2022), allergenic profilins have been identified not only in pollen but also in plant foods and latex [40]. Thus, profilin is considered to be responsible for the allergic cross-reactivity between these products [38]. Interestingly, it was also suggested that profilin might indicate allergy outcome and severity [41,42,43,44]. Moreover, naturally purified profilin may be used for the desensitization treatment of profilin-induced food allergy [45].

Other functional groups of proteins identified in bee pollen were transfer, resistance, response, and wall proteins. Moreover, we identified other proteins with allergenic activity. They were: pollen-specific protein Bnm1; Sal k 3 pollen allergen; Sal k 2; and pollen allergen MetE.

Pollen-specific protein Bnm1, taxonomically classified to *Brassica napus* (oilseed rape), contains peptide sites potentially allergenic. According to the computational studies, it was suggested that this protein is likely to be an allergen, although not included in the WHO/IUIS Allergen Nomenclature Database (www.allergen.org, accessed on 2 September 2022) [46]. It can be assumed that pollen-specific protein Bnm1 may be responsible for the allergenicity of both rapeseed and bee pollen of a specific origin. *Brassica napus* is a plant commonly cultivated in the area in Poland where the analyzed bee pollen was harvested. Hence, it is no surprise that rapeseed pollen was included in our bee pollen samples. However, we only identified the pollen-specific protein Bnm1 in yellow bee pollen samples. Yellow is the color of rapeseed flowers, as well as their pollen. Thus, our results confirm differences in the protein composition of bee pollen depending on the plant source.

Sal k 2 (protein kinase homolog) and Sal k 3 (cobalamin independent methionine synthase) are allergens of *Salsola kali* (Russian thistle, Saltwort), according to www.allergen.org (accessed on 2 September 2022). The allergenic properties of these proteins have been demonstrated elsewhere [47,48]. Pollen allergen MetE (cobalamin-independent methionine synthase) is a protein analogous to Sal k 3, also reported as being an allergen [49]. Interestingly, the abovementioned *Brassica napus* contain proteins sharing similarities with MetE [50]. Therefore, it should be noted that cross-reactivity can cause allergic reactions to bee pollen from different sources. There may be cases of an allergic reaction after ingesting bee pollen that does not contain the plant to which the consumer is allergic.

Among the proteins identified in this study are also hypothetical, uncharacterized, and unknown proteins. These proteins are of great interest because the functions and bioactivities of these structures are not yet characterized. Moreover, their expression has not been confirmed experimentally before. These proteins can affect the human body differently, with beneficial and harmful effects. Therefore, it is essential to characterize the unknown components of bee pollen. For this purpose, in the present study, the BLAST analysis (https://blast.ncbi.nlm.nih.gov/, accessed on 1 September 2022) on the identified hypothetical, uncharacterized, and unknown proteins was performed. With the BLAST tool, it is possible to find analogous regions in biological sequences of various plant species. This approach is designed to assess functional similarities between proteins in different species. The BLAST algorithm allows identifying members of protein families based on the nucleotide sequence alignment. The results of our analysis are presented in Supplementary Materials, Table S2.

Proteins identified with the BLAST approach were mostly enzymes. Many of these enzymes are involved in crucial developmental processes, such as energy metabolism or cell membrane and wall biosynthesis. They are, therefore, of colossal importance to plants. As mentioned earlier, many enzymatic proteins can have allergenic properties. However, to demonstrate such properties, additional tests must be performed.

In addition to the proteins taxonomically classified as *Viridiplantae*, in this study, we identified 10 proteins from the *Apis* spp. (see the Section 2, Table 1). Four of them were enzymes of carbohydrate metabolism: alpha-glucosidase; alpha-glucosidase III; glucose dehydrogenase [acceptor]-like; and glucose oxidase. These enzymes can help bees to process raw plant materials.

We also identified proteomic members of the major royal jelly proteins family and their precursors among *Apis* proteins. MRJP1 precursor was identified by the highest number of unique peptides in samples of all colors. MRJP1 is the most abundant protein belonging to the MRJPs family. Its other names are apalbumin or royalactin. This protein has been reported to possess mainly nutritional and developmental functions. Moreover, MRJP1 has antibacterial and antiproliferative activity [19,51,52]. MRJP2 and MRJP7, also identified in this study, have antioxidative and antibacterial properties [53,54]. They also show proliferative and migratory activities [55].

The MRJPs family also includes the proteins MRJP8 and MRJP9, classified as allergens. We did not detect these proteins in this study. However, their presence in bee pollen cannot be excluded. They may be present in other batches of bee pollen, collected at different times and from other sources. In order to rule out or confirm the presence of these allergens in the bee pollen, it would be necessary to test all batches of the product intended for consumption. That is why it is essential to develop indications for the standardization of bee pollen, which would increase the safety of its use.

The proteins produced by bees are an integral component of all bee products. Bees and their products are known for their allergenic properties. Although only 12 bee venom allergens are known to date, including MJRPs, there are likely more ingredients with such effects [56]. Therefore, people with diagnosed allergies, not just food allergies, should take extra care when consuming all bee-derived products, especially if they are trying a particular product for the first time.

Despite the risk of allergy after ingesting bee pollen, people who have not been diagnosed with an allergy should be keen to reach for it. Bee pollen is a high-protein product, but it also contains other valuable ingredients. Bee pollen is rich in carbohydrates such as sugars, polysaccharides, starch, and fibers. In addition, there can be found lipids, minerals, and a wide range of secondary plant metabolites (phytosterols, anthocyanins, vitamins, polyphenols, etc.) [14]. Due to these bioactive compounds, pollen offers several benefits for human health. Its bioactive constituents are involved in antioxidant, anti-inflammatory, antimicrobial, and immunostimulating activities. Some research also emphasized their potential anticancer properties [7,57]. Moreover, one study even showed bee pollen as a source of bacteria-producing antimicrobials [58]. Therefore, bee pollen has undoubted health benefits.

As bee pollen is a product obtained through biological processes, its composition and functionality can vary greatly. Qualitative reproducibility at the raw material collection stage is almost impossible. It is associated with the diverse ecosystem in which bees live and the highly complex botanical biodiversity. Hence, there is a critical need to develop guidelines for standardizing bee pollen, especially intended for consumption or therapeutic purposes.

## 4. Materials and Methods

### 4.1. Bee Pollen

Bee pollen samples included in the analyses were harvested in the summer of 2018 from a non-commercial apiary located in west-central Poland (Góry Złotnickie village, Calisia County, Greater Poland Voivodeship, 51°87′504″ N, 18°12′431″ E). The bee pollen pellets were visually divided into yellow, orange, violet, and brown colors. The color of the pellets depends mainly on the carotenoid and phenolic content. The samples analyzed in this study most likely contained compounds derived by bees from such plants as rapeseed (yellow pollen), phacelia (violet pollen), linden (yellow pollen), acacia (yellow and orange pollen), and fruit trees (mainly yellow, orange, and brown pollen). The surroundings of the apiary (within a radius of 20 km) are mostly fields where mainly the abovementioned plants are cultivated. In the area, there are also uncultivated lands and meadows. Therefore, the color of the bee pollen may indicate the primary plant source of the collected samples.

Pollen samples were collected using pollen traps. The traps were placed underneath the hive entrance, and returning foragers were required to walk through the dedicated plastic mesh to enter the hive. The mesh was specifically sized such that the foragers could pass through it, but large pollen loads were scraped off the bees’ rear legs and into a collection tray placed under the beehive bottom board [59].

### 4.2. Bee Pollen Samples Pre-Treatment

The fresh pollen samples were stored at −80 °C until pre-treatment and analyses. Then the samples were weighted and suspended in ultra-pure water at a 100 mg/mL concentration, each color separately. Each suspension was mixed on a laboratory shaker and ultrasonicated for 15 min in an ultrasonic bath. The mixtures were then centrifuged with 13,000 RPM (9600 RCF) for 10 min, and the supernatants were collected into separate tubes. Extracts were subjected to a relative protein enrichment using the equalizer technique.

A commercial *ProteoMiner*^TM^ Sequential Elution Small Capacity Kit (Bio-Rad, Hercules, CA, USA) was applied in the study. This kit was used to compress the dynamic range of protein levels in bee pollen extracts. The provided reagents contain a highly diverse library of hexapeptide ligands bound to chromatographic supports. Every single ligand of a given type can bind one unique protein. The number of ligands is limited. Hence, high-abundance proteins saturate their ligands, and protein excess is washed out. On the other hand, low-abundance proteins bind almost entirely to their specific ligands. This procedure equalizes the concentrations of all proteins present in the sample, thus preventing the masking of low-abundance proteins by high-abundance proteins.

The application procedure for the *ProteoMiner*^TM^ kit and subsequent solid-phase extraction and tryptic digestion steps is described in our previous article [52]. The sample solutions loaded onto the *ProteoMiner*^TM^ columns in this presented study were 0.2 mL supernatants (protein extracts) prepared as described above. Then all steps were performed according to the manufacturer’s instructions.

### 4.3. Analysis of a Total Protein Concentration in Bee Pollen Aqueous Extracts

Quantitative analysis of the total protein content of bee pollen aqueous extracts (before applying the *ProteoMiner*^TM^ kit) was performed using the Bradford method [60]. Aqueous extracts of bee pollen were spotted onto the microplate wells in a volume of 10 µL. Then 200 µL of Coomassie blue G-250 was added to each well. The plate was placed in a microplate reader Infinite F50 (Tecan, Männedorf, Switzerland), incubated for 5 min while agitating for 30 s, and the absorbance was measured at 595 nm. The calibration curve was plotted using standard (bovine serum albumin) solutions at concentrations of 1, 0.5, 0.125, and 0.0625 mg/mL.

### 4.4. Nanolc-MALDI-TOF/TOF MS Qualitative Proteomic Analyses

Proteins and peptides contained in bee pollen were subjected to identification using nanoLC-MALDI-TOF/TOF MS/MS technique. Every extract obtained from the *ProteoMiner*^TM^ depletion was analyzed once. There were four *ProteoMiner*^TM^ extracts from each sample. It means that four fractions in four separate runs were analyzed from one sample of each color. The first step of the proteomic identification involved the pre-treatment of the *ProteoMiner*^TM^ extracts with *ZipTip* C18 reverse-phase chromatography pipette tips. For the nanoLC separation, the nanoflow HPLC (high-performance liquid chromatography) system (Easy-nLC II, Bruker Daltonics, Bremen, Germany) and fraction collector (Proteineer-fc II, Bruker Daltonics, Bremen, Germany) were utilized. For the protein and peptide concentration, NS-MP-10 BioSphere C18 (NanoSeparations, Nieuwkoop, the Netherlands) column was used, and an Acclaim PepMap 100 (Thermo Scientific, Sunnyvale, CA, USA) column (150 mm × 75 μm I.D.m particle size μm, pore size 100 Å) was used for the separation. The parameters of the linear gradient elution method were as follows: 2–50% ACN (acetonitrile) in 96 min (mobile phase A: 0.1% TFA in water; mobile phase B: 0.1% TFA in ACN). The linear gradient elution was applied at the flow rate of 300 nL/min, and 3 μl of the sample eluent was injected into the chromatography column. As the result of the separation process, 384 fractions were collected, and each fraction was further mixed with a matrix solution (36 μL of HCCA saturated solution in 0.1% TFA and ACN (90:10 *v/v*), 748 μL of ACN and 0.1% TFA (95:5 *v/v*) mixture), 8 μL of 10% TFA, and 8 μL of 100 mM ammonium phosphate and then spotted automatically onto AnchorChip 384 MALDI target plate (Bruker Daltonics, Bremen, Germany) using the fraction collector. For the MS/MS analysis of the collected fractions, UltrafleXtreme (Bruker Daltonics, Bremen, Germany) mass spectrometer was used. The MS/MS analysis was held in a mass range *m/z* of 700–3500. The mixture of Peptide Calibration Standard II (Bruker Daltonics, Bremen, Germany) was used for the external calibration. The control software for the nanoLC was HyStar 3.2 (Bruker Daltonics, Bremen, Germany). The precursor ions for the identification were set using WARP-LC (Bruker Daltonics, Bremen, Germany) software. MALDI-TOF MS instrument was controlled by FlexControl 3.4 (Bruker Daltonics, Bremen, Germany) software. For the spectra processing and evaluation, FlexAnalysis 3.4 and BioTools 3.2 (Bruker Daltonics, Bremen, Germany) software were used. The identification of discriminative peptides and proteins was performed using the ProteinScape 3.1 and Mascot 2.4.1 search engine. *Apis* spp. and *Viridiplantae* were applied as a taxonomic restriction with the following protein search parameters: precursor ion mass tolerance ± 50 ppm; fragment ion mass tolerance *m/z* ± 0.7; monoisotopic mass; peptide charge +1.

## 5. Conclusions

At present, strong trends can be observed in the food industry about the abandonment of highly processed products in favor of products of natural origin, whose valuable biological properties and the safety of their use have been known for centuries. Such conditions are met by bee pollen, the so-called “superfood”. Bee pollen can contain up to 23% protein, depending on the botanical origin. This high protein content means that the extract from this raw material can act as a new dietary supplement of particular interest to vegetarians. However, for a product to be safe, it must be thoroughly characterized for allergens and other ingredients with potentially harmful effects. Therefore, in this study, we characterized the proteomic composition of bee pollen collected in Poland. We identified 207 unique proteins, among which were several allergens (calmodulins, profilin, pollen-specific protein Bnm1, Sal k 3 pollen allergen, Sal k 2, pollen allergen MetE, and MRJPs). The most interesting identified proteins are hypothetical, uncharacterized, and unknown proteins. The functions and bioactivities of these proteins are not yet characterized, and their expression has not been confirmed experimentally before. By using bioinformatics tools, we identified these proteins mainly as enzymes. They may have potential allergenic activity, which should be confirmed in further experiments.

This study is the essential step toward characterizing bee pollen proteomic composition. The presented results depict the allergens contained in this nutritious product. These findings draw attention to the fact that the consumption of bee pollen, in addition to its undoubted health benefits, may carry the danger of allergies (including anaphylaxis) in sensitized individuals. Therefore, this work is of particular importance because public awareness of the allergen content of bee pollen and other bee products can prevent health- or life-threatening incidents following the ingestion of these increasingly popular “superfoods”.

## Figures and Tables

**Table 1:** Proteins identified in bee pollen classified to *Apis* spp.; [1]—*Apis cerana indica*; [2]—*Apis dorsata*; [3]—*Apis mellifera*; [4]—*Apis cerana cerana*; [5]—*Apis florea*; [6]—*Apis cerana*.

Accession	Protein Name	Number of Peptides Identified as a Protein Fragment				Classes/Functions
		Orange	Violet	Yellow	Brown	
gi|126842411	alpha-glucosidase [1]				3	Carbohydrate metabolism [17]
gi|283105164	alpha-glucosidase III [2]				3	Carbohydrate metabolism [17]
gi|284812514	MRJP5 [3]	6				Nutrition and development [19]
gi|33358394	major royal jelly protein MRJP1 [4]	7				Nutrition and development [19]
gi|380025661	PREDICTED: glucose dehydrogenase [acceptor]-like [5]		3		3	Carbohydrate metabolism [18]
gi|40557703	major royal jelly protein MRJP1 precursor [6]	11	8			Nutrition and development [19]
gi|58585090	glucose oxidase [3]	5	15	6	6	Carbohydrate metabolism [20]
gi|58585098	major royal jelly protein 1 precursor [3]	82	57	40	39	Nutrition and development [19]
gi|58585108	major royal jelly protein 2 precursor [3]	4		4	5	Nutrition and development [19]
gi|62198227	major royal jelly protein 7 precursor [3]	11				Nutrition and development [19]

**Table 2:** Total protein concentration in aqueous solutions of bee pollen harvested in June and July 2018; SD—standard deviation; RSD—relative standard deviation.

Sample No	Harvest Date	Proteins Concentration [mg/mL]
1	14 June 2018	0.91252
2	23 June 2018	0.97429
3	8 July 2018	0.73127
4	20 July 2018	0.68932
Mean		0.82685
SD		0.137995096
RSD		16.68925395

## Data Availability

The data presented in this study are contained within the article.

## Supplementary Materials

The following supporting information can be downloaded at: https://www.mdpi.com/article/10.3390/molecules27227733/s1, Table S1: Proteins of Viridiplantae; Table S2: Results of BLAST analysis.

## Sample Availability

Samples of the compounds are available from the authors.
