# Supplementary material for: Mass Spectrometry-Based Identification of Bioactive Bee Pollen Proteins: Evaluation of Allergy Risk after Bee Pollen Supplementation
Source: Molecules. 2022 Nov 10;27(22):7733. doi: 10.3390/molecules27227733 (PMC9695670; doi:10.3390/molecules27227733)
Supplement: Supplementary file 1 [file molecules-27-07733-s001.zip › Molecules-1993749-Supplementary Materials Table S2 - Results of BLAST analysis.pdf]

molecules

Supplementary Materials

Mass Spectrometry-Based Identification of Bioactive Bee Pollen Proteins: Evaluation of Allergy Risk After Bee Pollen Supplementation

Eliza Matuszewska [1,*], Szymon Plewa [1], Dagmara Pietkiewicz [1], Kacper Kossakowski [1], Joanna Matysiak [2], Grzegorz Rosiński [3] and Jan Matysiak [1]

Table S2. Results of BLAST analysis including the highest sequence alignments to characterised proteins. [1] - Vitis vinifera; [2] - Arabidopsis lyrata subsp. lyrate; [3] - Glycine max; [4] - Brachypodium distachyon; [5] - Arabidopsis thaliana; [6] - Brassica rapa; [7] - Zea mays; [8] - Solanum tuberosum; [9] - Vitis riparia; [10] - Capsella rubella; [11] - Oryza sativa Japonica Group; [12] - Glycine soja; [13] - Solanum verrucosum.

Accession	Protein Name	Significant Alignment Sequence	Query Coverage	Percent Identity	E-Value
gi|147780898	Hypothetical protein VITISV_007504 [1]	polyprotein [1]	57%	79%	0.0
gi|147815609	hypothetical protein VITISV_036160 [1]	PREDICTED: UDP-arabinopyranose mutase 3 [1]	90%	100%	0.0
gi|147821107	hypothetical protein VITISV_018473 [1]	5-methyltetrahydropteroyltriglutamate--homocysteine methyltransferase [9]	100%	95%	0.0
gi|297796049	hypothetical protein ARALYDRAFT_495305 [2]	actin-depolymerizing factor 12 [10]	100%	100%	2×10^{-96}
gi|297804932	hypothetical protein ARALYDRAFT_915516 [2]	adenosylhomocysteinase 1 [2]	100%	100%	0.0
gi|297805822	hypothetical protein ARALYDRAFT_916391 [2]	UDP-glucose 6-dehydrogenase 4 [2]	100%	100%	0.0
gi|297811689	hypothetical protein ARALYDRAFT_488394 [2]	UDP-glucose 6-dehydrogenase 3 [2]	100%	100%	0.0
gi|297812139	hypothetical protein ARALYDRAFT_488852 [2]	aldehyde oxidase GLOX1 [2]	100%	100%	0.0
gi|297820020	hypothetical protein ARALYDRAFT_906673 [2]	fructose-bisphosphate aldolase 8, cytosolic [2]	100%	100%	0.0
gi|297827125	hypothetical protein ARALYDRAFT_482625 [2]	fructose-bisphosphate aldolase 6, cytosolic [2]	100%	100%	0.0
gi|297829230	hypothetical protein ARALYDRAFT_478006 [2]	peroxisomal fatty acid beta-oxidation multifunctional protein MFP2 isoform X2 [2]	100%	100%	0.0
gi|297831264	hypothetical protein ARALYDRAFT_898993 [2]	late embryogenesis abundant protein 1 [2]	100%	95%	2×10^{-52}
gi|297832836	hypothetical protein ARALYDRAFT_896165 [2]	UDP-glucose 6-dehydrogenase 3 [2]	100%	100%	0.0

gi|297833574	hypothetical protein ARALYDRAFT_478121 [2]	polygalacturonase isoform X1 [2]	100%	100%	0.0
gi|297834164	hypothetical protein ARALYDRAFT_478720 [2]	L-ascorbate oxidase homolog [2]	100%	100%	0.0
gi|297839613	hypothetical protein ARALYDRAFT_476916 [2]	protein disulfide isomerase-like 1-2 [2]	97%	100%	0.0
gi|297841411	hypothetical protein ARALYDRAFT_475824 [2]	aldehyde oxidase GLOX1 [2]	100%	97%	0.0
gi|351720697	uncharacterized protein LOC100499870 [3]	NAD(P)H dehydrogenase (quinone) FQR1-like isoform 1 [3]	100%	100%	1×10^{-133}
gi|356574489	PREDICTED: uncharacterized protein LOC100783153 [3]	mediator of DNA damage checkpoint protein 1 isoform X2 [3]	77%	96%	0.0
gi|357117399	PREDICTED: uncharacterized protein LOC100827223 [4]	hapless 2 isoform X2 [11]	100%	64%	6×10^{-54}
gi|363807140	uncharacterized protein LOC100795446 [3]	Fructose-bisphosphate aldolase 6, cytosolic-like [3]	100%	100%	0.0
gi|116831345	unknown [5]	pfkB-like carbohydrate kinase family protein [5]	99%	100%	0.0
gi|119720822	unknown [6]	late embryogenesis abundant protein 2 [6]	100%	100%	5×10^{-71}
gi|238015186	unknown [7]	glyceraldehyde-3-phosphate dehydrogenase 1, cytosolic isoform X2 [7]	100%	100%	0.0
gi|255629031	unknown [3]	Monodehydroascorbate reductase isoform B [12]	100%	100%	3×10^{-120}
gi|255638733	unknown [3]	UDP-glucose 6-dehydrogenase 1 [3]	100%	100%	0.0
gi|255638991	unknown [3]	probable UDP-arabinopyranose mutase 1 [3]	98%	100%	0.0
gi|77999255	unknown [8]	fructose-bisphosphate aldolase 6, cytosolic [13]	100%	100%	0.0
